# The Impact of Citizenship Pressure on Organizational Citizenship Performance: A Three-Way Interactive Model

**DOI:** 10.3389/fpsyg.2021.670120

**Published:** 2021-09-30

**Authors:** Yazhen Liu, Jingtao Fu, Sabeeh Pervaiz, Qi He

**Affiliations:** ^1^School of Management, Hainan University, Haikou, China; ^2^Institute of Corporation Governance Research of Hainan Province, Haikou, China; ^3^School of Management, Jiangsu University, Zhenjiang, China; ^4^School of Finance and Economics, Jiangsu University, Zhenjiang, China

**Keywords:** citizenship pressure, organizational citizenship performance, transformational leadership, political skill, expectancy theory

## Abstract

Citizenship pressure has recently been a hot topic in organizational citizenship behavior research since it aids in understanding the driving mechanism of organizational citizenship behavior. However, previous research has revealed discrepancies in the connection. This article develops a theoretical model of the impact of citizenship pressure on organizational citizenship performance based on expectancy theory. A leader–employee paired questionnaire was used to evaluate the hypotheses. The results indicate that organizational citizenship performance is positively influenced by citizenship pressure. The connection between organizational citizenship performance and citizenship pressure is positively moderated by transformational leadership. The better the transformational leadership, the greater the influence of citizenship pressure on organizational citizenship performance. Furthermore, the impact of transformational leadership on the link between citizenship pressure and organizational citizenship performance is dependent on the political skill of employees. When individuals with strong political skill encounter transformational leadership, the relationship between citizenship pressure and organizational citizenship performance is minimal. On the contrary, this relationship is enhanced when personnel with limited political skill are confronted with transformational leadership.

## Introduction

Coronavirus disease 2019 (COVID-19) has triggered many challenges and crises for organizations. When confronted with a disaster, companies require employees to contribute more to the organization in order to weather the storm. Especially in this day of “doing more with less,” the need for companies to accomplish more with less is becoming increasingly apparent. With their organizational citizenship behavior (OCB), more organizations are hoping to enhance organizational growth without raising costs (Zhao et al., 2014; Zhang et al., 2020). Organizations expect workers to fulfill their tasks and to conduct outside their jobs (Zhou and Long, 2012). However, when employees engage in OCB, the unique needs of organizations will drain their energy and resources and make them feel a certain strain (Zhao and Jiang, 2017). Employees experience citizenship pressure when their organizations and leaders want them to engage in more OCBs (Bolino et al., 2010). Too much pressure on workers, for instance, OCB, is generally believed to diminish their good conduct. However, this is not the case; employees continue to engage in positive behaviors even when they are stressed.

Organizational citizenship behavior was established as employee-driven conduct, including arbitrary work-related behavior, independent of the official organizational incentive structure and it enhances the overall effectiveness of organizations (Smith et al., 1983; Zhang et al., 2020). However, with rising market competitiveness, especially in present complex and fast-paced businesses, OCB has progressively evolved into an expectation of the organization and leaders from employees. The behavior of employees outside their anticipated tasks has become essential to the success of organizations. OCB of employees has been considered as a key source of the sustained competitive advantage of firms (Podsakoff et al., 2009). Performance and behavior are linked because behavior causes performance, and only behavior may escalate performance. Organizational citizenship performance (citizenship behavior related to the organization) is a type of OCB discussed in the next phase of the study (Podsakoff et al., 2000). But this study focuses on organizational citizenship performance. Because organizational citizenship performance is a citizenship behavior that is conducive to organizational performance as a whole and points to the organizations, some studies on OCB show that OCB is not necessarily beneficial to both employees and organizational performance (Liu et al., 2017), pointing to individuals and organizations. Previous research has produced contradictory findings of the relationship between citizenship pressure and OCB. In some researches, both are associated positively (Cates et al., 2010); however, in others, researchers state that they have a negative correlation or no significant relationship (Zhao et al., 2014). As a result, this research aims to ascertain why employees continue to participate in OCB even when they are under pressure and understand the relationship between them.

The moderating impact of transformational leadership and political skill of employees is examined in this study. Transformational leadership moderates the relationship between citizenship pressure and organizational citizenship performance. On the contrary, the moderating variable of transformational leadership is moderated by political skill. Seeing as leaders is important in every company, and leadership style impacts the culture and future development of organizations. Transformational leadership has received much attention in recent decades, notably in OCB (Christoph and Guido, 2017). Employees are more likely to participate in virtuous activities when their leaders are transformational (Burns, 1978). As a result, leadership style matters, and OCB is worth investigating. It is worth understanding the process for transformational leadership to get outcomes that surpass expectations through the management strategy, promoting extra-role behaviors by workers. China is a society that stresses personal relations, so the interpersonal relationship is important in Chinese companies for day-to-day work and living. As a result, in this unique organizational setting, personnel with high political skill can carry out interpersonal interaction in a variety of situations due to their exceptional social ability and acute understanding. It will improve communication with leaders by making a good impression on leaders by showing sincerity and establish a good personal relationship with transformational leadership (Ferris et al., 2007), lowering the expectations of their leaders of their OCB (Tian and Yang, 2019 ) and becoming refined egoists who can meet the expectations of their leaders by doing less OCB (Ferris et al., 2007). On the contrary, employees with strong political skill may remain cool under pressure, manage their surroundings, and experience stresses in ways that mitigate the negative impacts of pressure and minimize the production of OCB (Ferris et al., 2007).

The moderating impact of transformational leadership was first investigated by considering transformational leadership as a factor influencing the attitude and behavior of employees. Second, we investigated whether the political skill of workers will impact the function of transformational leadership, as well as the link between citizenship pressure and organizational citizenship performance. Finally, this study constructs a regulated regulatory model between citizenship pressure and organizational citizenship performance based on expectancy theory to explore the link between the two and their model parameters. According to this study, employees will engage in OCB pointed by citizenship pressure out of their good expectations for the future (expectancy), so as to meet the requirements of the organization, and achieve valuable (valence) results (instrumentality). This study aims to tackle the contradictory link between citizenship pressure and organizational citizenship performance in prior studies while using the model. On the contrary, research on citizenship pressure can assist organizational managers in better understanding their employees, timely alleviate citizenship pressure, reduce its negative impact on employees, and guide them to engage in positive behavior, which can serve as a model for future practice.

## Literature Review and Research Hypotheses

### The Impact of Citizenship Pressure on Organizational Citizenship Performance

The continual physical and psychological (cognitive or emotional) efforts of employees are referred to as job need (Schaufeli and Bakker, 2004). Previous research on job need focuses largely on role overload and working hours. Citizenship pressure is a new research direction in the field of job need. Organizations often convey to employees the expectation of engaging in more OCBs through various means, such as organizational norms, stating the behaviors of excellent employees, emphasizing the deeds of model employees, and so on, which puts pressure on employees when performing OCBs (Bolino et al., 2010). The term citizenship pressure refers to the subjective perceptions of employees of how much pressure they are under to engage in the so-called voluntary OCB. Pressure not only leads to negative outcomes, such as stress in the workplace (Bolino et al., 2010), resignation intention, work–family conflict, and diminished happiness (Bolino et al., 2013). It also has a beneficial effect on family–work gains (Cates et al., 2010), employment, professional growth, social exchanges, and other consequences (Cates et al., 2010).

As a variable with the most evident relationship with citizenship pressure, organizational citizenship performance refers to citizenship behaviors that are highly beneficial to the organization as a whole, such as complying with the organization and defending the organization. While OCB has historically been conceptualized as the discretion of behavior of an individual, it has recently taken on non-voluntary aspects, i.e., these extra-role behaviors are not spontaneous and active behaviors of employees, but forced OCBs of employees under certain circumstances (Vigoda-Gadot, 2006). Therefore, the execution of OCB outlined in this article is not discretionary behavior whatsoever, but rather the expectations and needs of organizations.

Citizenship pressure, according to this study, might inspire workers to engage in more OCBs. The following are the reasons: there are two types of pressure that people face at work: positive pressure and negative pressure. People who hope to achieve by overcoming pressure are said to be under positive pressure. On the contrary, negative pressure is detrimental to the growth of individuals and the development of the company (Tian and Yang, 2019). According to expectancy theory, when faced with positive pressure, people generally assume that their efforts will result in comparable returns. By skillfully managing with pressure, they can reap beneficial rewards (Lepine et al., 2005). Workplace rivalry is getting increasingly intense as employment pressures rise. Employees think that taking on additional duties and engaging in more OCBs will help them enhance their performance, compensation, and job satisfaction. As a result, they will consider citizenship pressure to be a form of positive pressure. To put it another way, when companies tell their employees that they expect them to engage in greater OCBs, their citizenship pressure rises. Employees will engage in greater OCBs as a result of this pressure (Tian and Yang, 2019). Therefore, employees will take the initiative to address citizenship pressure areas if they have realistic expectations for their own growth and professional success. To relieve the current level of citizenship pressure, employees need to engage in more OCBs. We offered the following hypothesis based on the aforementioned investigation:

**Hypothesis 1:** Citizenship pressure has a positive impact on the organizational citizenship performance of employees.

### The Moderating Role of Transformational Leadership

Transformational leaders are people who have strong inherent influences and charisma. They are accessible and treat employees well. They look after individuals, helping them create life and work objectives, improving intrinsic motivation, and inspiring their inner high-level needs (Bass, 1985; Bass et al., 1987). They motivate people to put the interests of organizations ahead of their own and put more effort (Avolio et al., 1999) to attain more significant organizational goals (Wu et al., 2009). Understanding transformational leadership is required to understand why employees work so hard. Transformational leadership increases the awareness and understanding of ethical values and inspiring visions of employees, which significantly increases the satisfaction of employees with the organization. They encourage employees to go beyond personal goals and interests for the benefit of the group (Howell and Shamir, 2005), and motivate employees to achieve performance beyond the expected level (Avolio et al., 2004).

According to the social exchange theory, all human behaviors are driven by explicit or implicit trade activities that might result in rewards (Birtch et al., 2016). As a result, all social activities and certain social connections created as a result of social interaction can be summarized as an exchange (Emerson, 1976). We assume that transformational leadership and employees have a social exchange connection (Graen and Uhl-Bien, 1995). Citizenship pressure has a greater beneficial influence on OCB when high-level transformational leadership is involved. First and foremost, transformational leaders inspire, assist, and give individualized attention to employees (Bass and Avolio, 1990), and employees will feel more obligated to repay their leaders. Employees will see engaging in more OCBs as a means of repaying their leaders under the impact of transformational leadership. In contrast, citizenship pressure can drive employees to participate in OCB. As a result, when employees are faced with citizenship pressure, they will put in additional effort at work, invest more emotions and practical actions in the company, and generate citizenship behaviors that are helpful to the organization in order to reward their leaders.

Second, transformational leadership will amplify the benefits of citizenship pressure on the OCB of employees. Transformational leadership considers how to arouse the emotions and motivation of employees, to stimulate their reasonable expectations for the future, and to make employees realize the importance and value of their work (Farh et al., 2006). They also encourage employees to see citizenship pressure as a positive pressure, to boost the confidence of employees in overcoming citizenship pressure and achieving their goals (Wu and Wu, 2007; Zhang and Wang, 2017). To reduce the existing citizenship pressure, employees should adopt a more proactive strategy for dealing with citizenship pressure. They should give their time and energy to the business and engage in more OCBs. We proposed the following hypothesis based on the above analysis:

**Hypothesis 2:** Transformational leadership positively moderates the relationship between citizenship pressure and organizational citizenship performance.

### The Moderating Role of Political Skill

The capacity to successfully understand the motives of people for work behaviors and to utilize the knowledge and experience of an individual to encourage others to act in ways that achieve personal and organizational goals is referred to as political skill (Ferris et al., 2005). It has four dimensions: social astuteness, interpersonal influence, networking ability, and apparent sincerity. The capacity of individuals to adapt to diverse social settings and properly explain the conduct of their own and others in the social context is referred to as social astuteness. Interpersonal influence ability refers to the ability of individuals to adjust their behavior in order to influence others. Networking ability refers to the ability of individuals to create and use various interpersonal networks in order to put themselves in a better position. Apparent sincerity refers to the ability of individuals to gain the trust of others by demonstrating sincerity (Ferris et al., 2005). The study of the antecedents of political skill focused on the personality of individuals and social environment variations (Ferris et al., 2007). The assessments of the organizational environment of individuals, the molding of values of individuals, and the responses of individuals to the environment all play a part in political skill (Ferris et al., 2005). In general, political skill assists individuals in adapting their actions to various situations in order to get a competitive edge at work (Andrews et al., 2009). As a result, the behaviors of individuals will change depending on their level of political skill (Chen et al., 2021).

According to this study, the impact of transformational leadership on citizenship pressure and organizational citizenship performance is diminished when people have a high degree of political skill. The following are the reasons: on the one hand, when faced with citizenship pressure, employees with high political skill have an exceptional social skill and a strong environmental perception and can engage in flexible interpersonal interaction in response to situational changes (Wu et al., 2012). They are more likely to have opportunities to interact with and collaborate with transformational leaders and foster mutual understanding. These interactions, in turn, afford employees opportunities to enhance their personal relationships with leaders, to build trust, and to generally contribute to favorable impressions formed by the leader about them (Braendle et al., 2005; Ferris et al., 2007; Wu and Yang, 2019). This reduces the expectations of transformational leaders of employees in their OCBs (Douglas and Ammeter, 2004). As a consequence, they do not participate in “too much” OCB or transcend the present stage of citizenship pressure (Tian and Yang, 2019), resulting in the impact of “getting twice the outcome with half the work” (Tian and Yang, 2019); on the other hand, political skill helps employees to remain calm and confident, reduce tension, and thus control the surrounding environment. When faced with pressure from transformational leadership, high-political-skilled employees can mitigate the negative impacts of pressure by how they perceive and interpret them (Zhao, 2014). The pressure of transformational leaders is seen to be less stressful (Ferris et al., 2007), which reduces the creation of OCB. We suggested the following hypothesis based on the relevant study:

**Hypothesis 3:** Political skill negatively moderates the moderating impact of transformational leadership on the relationship between citizenship pressure and organizational citizenship performance.

**Figure 1:**
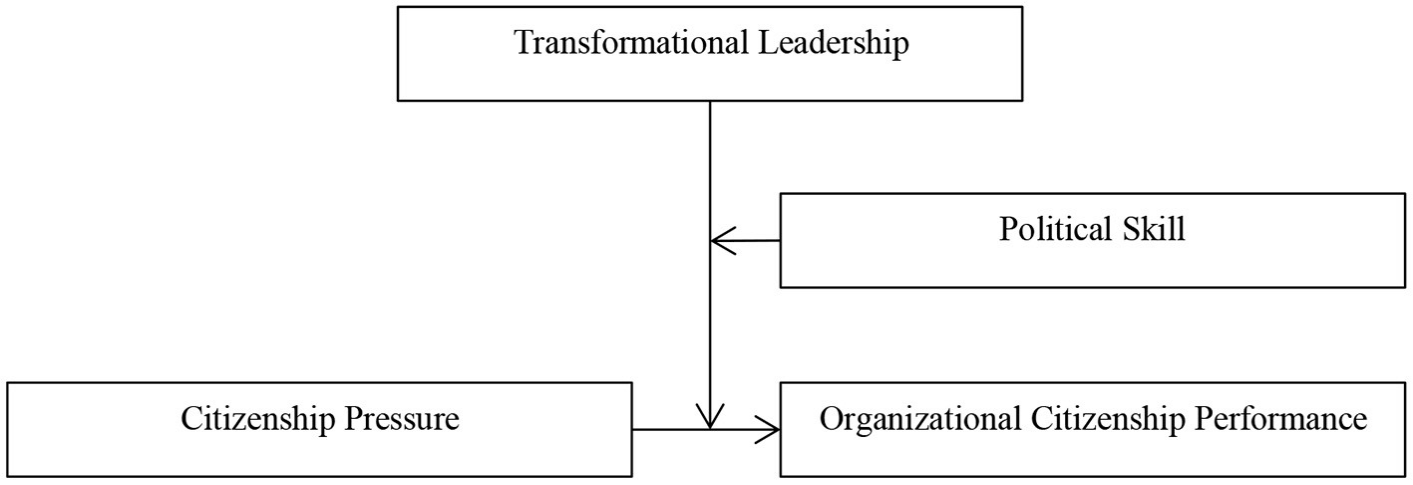
Theoretical model.

Therefore, the theoretical model of this study is shown in Figure 1.

## Methodology

### Data Collection

The data for this study were collected using a questionnaire survey from various organizations in Hainan, China. They are widely distributed in Haikou and surrounding cities and counties. Among the 10 organizations participating in the survey, there are relatively more public enterprises, and the construction industry, financial industry, and sales industry account for a large proportion. The specific information is shown in Table 1. A leader–employee pairing sample was gathered to prevent the common method deviation. To undertake questionnaire surveys, we have chosen specific organizations in which we have solid ties and partnerships. Initially, we contacted the human resources managers of target companies. Once we received their consent, we scheduled a time with them and gathered the staff in the conference room. The participants were assured that the survey data would be kept anonymous and would only be used for academic purposes. To help employees understand the questionnaire, we discussed each question on the spot and then asked them to fill out the questionnaire.

**Table 1:** Sample distribution characteristics of organizations.

**Organization number**	**Type of organization**	**Industry**	**Number of participants /total number of employees**	**Sector**
1	Public enterprises	Construction	37/270	Technical, Finance, Business, Purchasing, Engineering, Administrative
2	Public enterprises	Financial	32/214	Technical, Finance, Business, Operation, Administrative
3	Public enterprises	Financial	36/265	Technical, Finance, Business, Operation, Administrative
4	Public enterprises	Sales	33/231	Technical, Finance, Business, Operation, Administrative
5	Public enterprises	Construction	40/306	Technical, Finance, Business, Purchasing, Engineering, Administrative
6	Public enterprises	R and D	31/256	Technical, Finance, Business, Operation, Administrative, Strategic
7	Public enterprises	Petrochemical	38/328	Technical, Finance, Operation, Administrative
8	Private enterprises	Sales	21/130	Finance, Business, Operation, Administrative
9	Private enterprises	Financial	23/151	Finance, Business, Operation, Administrative
10	Private enterprises	Sales	20/127	Finance, Business, Operation, Administrative

In this process, some employees felt sensitive and withdrew from the survey. After receiving the questionnaire, some of the responses did not match and had a strong preference, so they were eliminated. Both employee and leader respondents used paper-based questionnaires. Employees themselves evaluated their citizenship pressure, political skill, and transformational leadership, and leaders evaluated the organizational citizenship performance of employees. Initially, 350 questionnaires were provided, with 297 valid questionnaires being matched, resulting in an effective recovery rate of 84.86%. This article examines the impact of citizenship pressure experienced by employees on their organizational citizenship performance, so the sample subject is employees. Table 2 shows the characteristics of the effective sample distribution of employees.

**Table 2:** Sample distribution characteristics of employees.

**Variable**	**Category**	**Proportion**
Gender	Male	56.30%
	Female	43.70%
Age	≤ 20	18.20%
	21~30	50.00%
	31~40	16.60%
	41~50	13.80%
	>50	1.40%
Education	Senior high school education and below	12.80%
	Junior college education	30.10%
	Bachelor's degree	53.00%
	Master's degree or above	4.10%
Working years in the current organization	<1	16.20%
	1~3	8.80%
	3~5	5.40%
	5~10	34.80%
	>10	34.80%
Type of work organization	State-owned enterprises	83.10%
	Private enterprises	16.90%
	The rest	0%
Type of occupation	Management	20.60%
	Technology	72.30%
	Business	1.40%
	Creative design	0%
	The rest	5.70%

This study adopts a leader–employee pairing sample. The leaders evaluate the organizational citizenship performance of the employees to make the study more rigorous and objective. Although employees are the subject of the study, it is still necessary to investigate the leaders. Therefore, we describe the characteristics of the effective sample of leaders and fully explain its rationality. In the effective sample, male leaders accounted for 58.2% and female leaders 41.8%. The majority of leaders aged 40 and above; 83.2% of leaders with a bachelor's degree or higher. Grassroots leaders accounted for 26.6%, middle-level leaders accounted for 52.1%, and senior leaders accounted for 21.3%.

### Measurement Tools

This study derived scales from previously tested scales, either directly or indirectly. The English scales were translated and back-translated using the technique suggested by Brislin (1980). To create the final Chinese scales, the linguistic expression and narration of the scale were changed. The Likert seven-point scoring method was used to evaluate all replies.

**Citizenship pressure:** This study used the eight-item scale of citizenship pressure scale developed by Bolino et al. (2015). Cronbach's reliability coefficient of 0.901 approves the reliability of this scale. One of the items states, “In my department, taking on additional responsibilities and volunteer for additional tasks will bring me a lot of pressure.”

**Transformational leadership:** The eight-item scale of transformational leadership scale developed by Chen et al. (2006) was used in this research. One of the scale items states, “My leaders often describe an encouraging future to us.” Having Cronbach's reliability coefficient of 0.882 expresses the high reliability for this scale.

**Political skill:** The political skill scale developed by Ahearn et al. (2004) was used for this study. This scale has six items, and the Cronbach's reliability coefficient is 0.775. One of the scale items states, “I'm good at building good relationships with most people.”

**Organizational citizenship performance:** The seven items organizational citizenship performance scale developed by Williams, 1991 was used in this study. This scale has Cronbach's reliability coefficient of 0.788. One of the scale items states, “He/she will save and protect the property of the organization.”

**Control variables:** This study selected demographic variables that may affect citizenship pressure, transformational leadership, political skill, and organizational citizenship performance. These variables mainly include gender, age, education level, working years, organizational type, and occupation type.

### Analysis Procedure

Harman single factor analysis was used to do exploratory factor analysis on all four variables in this investigation to see if there were any common method bias problems. Then, using AMOS 24.0, confirmatory factor analysis was done on the survey data to assess the matching between the data, model, and discriminant validity of the variables. We conducted descriptive statistical analysis on the control variables and the four variables to assess the correlation between the variables after confirming that the data did not have severe common method bias issues and had high discriminative validity among the four variables. Second, each hypothesis is tested using the hierarchical regression test suggested by Baron and Kenny, with diagrams produced to illustrate the moderating effects clearly.

## Data Analysis

### Common Method Deviation Test and Confirmatory Factor Analysis

This research adopted Harman single factor analysis to carry out exploratory factor analysis for all the items across the four variables. The results showed that KMO = 0.711, the chi-square value of spherical test suggested by Bartlett was 8,734.973, and the *p*-value was <0.001. The four common factors extracted were consistent with the number of variables set in this study. The first-factor variability was 18.87%, lower than the critical value of 50%, so the data were deemed not to have serious common method bias issues. Besides, all data were obtained anonymously, and the research procedures were strictly controlled to control for common method deviation.

AMOS 24.0 was used to test the discriminant validity of the variables. The results of the confirmatory factor analysis are shown in Table 3. Compared with other models, the four-factor model fit the index best (χ^2^/df = 2.564, RMSEA = 0.073, CFI = 0.934, IFI = 0.934, TLI = 0.927). This confirmed the factors of citizenship pressure, transformational leadership, political skill, and organizational citizenship performance having good discriminative validity.

**Table 3:** Confirmatory factor analysis.

**Model**	**χ^2^**	**df**	**χ^2^/df**	**RMSEA**	**CFI**	**IFI**	**TLI**
Four factors D	941.143	367	2.564	0.073	0.934	0.934	0.927
Three factors C	3501.688	371	9.439	0.169	0.638	0.640	0.604
Two factors B	4977.218	374	13.308	0.204	0.468	0.470	0.422
One factor A	1265.776	377	3.357	0.089	0.120	0.145	0.052

*(A) Citizenship pressure + transformational leadership + political skill + organizational citizenship performance; (B) Citizenship pressure + transformational leadership + political skill; organizational citizenship performance; (C) Citizenship pressure, transformational leadership + political skill, organizational citizenship performance; (D) Citizenship pressure; transformational leadership; political skill; organizational citizenship performance*.

### Descriptive Statistics and Correlation Analysis

Table 4 mainly shows the mean, standard deviation, and correlation coefficient of each variable. As can be seen from Table 4, there is a significant positive correlation between citizenship pressure and organizational citizenship performance (*r* = 0.236, *p* < 0.001). That is, citizenship pressure has a positive effect on organizational citizenship performance. There is a significant positive correlation between transformational leadership and organizational citizenship performance (*r* = 0.222, *p* < 0.001). Furthermore, there is a significant correlation between political skill and organizational citizenship performance (*r* = −0.190, *p* < 0.01). This offers a foundation for the research hypothesis of this study to be tested further.

**Table 4:** Descriptive statistics and correlation analysis.

**Variable**	**Mean**	**SD**	**1**	**2**	**3**	**4**	**5**	**6**	**7**	**8**	**9**	**10**
**(1) Gender**	1.20	0.400	1									
**(2) Age**	2.30	0.966	0.038	1								
**(3) Education**	2.48	0.767	−0.084	−0.393^***^	1							
**(4) Working years**	3.63	1.442	−0.089	0.725^***^	−0.225^***^	1						
**(5) Organization type**	1.17	0.375	0.340^***^	0.289^***^	−0.391^***^	0.122^*^	1					
**(6) Occupation type**	1.98	0.858	0.160^**^	−0.082	−0.139^*^	−0.197^**^	0.168^**^	1				
**(7) Citizenship pressure**	3.37	0.578	0.020	0.093	−0.055	0.229^***^	−0.048	−0.045	1			
**(8) Transformational leadership**	4.25	0.459	−0.046	0.013	−0.026	0.046	−0.044	0.034	−0.007	1		
**(9) Political skill**	4.37	0.411	−0.003	−0.005	−0.018	0.067	0.091	−0.014	−0.058	−0.020	1	
**(10) Organizational citizenship performance**	4.04	0.430	−0.092	0.073	−0.035	0.109	0.037	−0.023	0.236^***^	0.222^***^	−0.190^**^	1

* *p < 0.05*,

** *p < 0.01*,

*** *p < 0.001*.

### Hypothesis Testing

To test the moderating effects of transformational leadership and political skill on the realationship between citizenship pressure and organizational citizenship performance, this research first standardized variables and then used the hierarchical regression test method suggested by Baron and Kenny (1986) to test Hypotheses 1–3. Table 5 shows the results.

**Table 5:** Regression analysis results.

**Variable**	**Organizational citizenship performance**			
	**Model 1**	**Model 2**	**Model 3**	**Model 4**
**Control variable**				
Gender	−0.112	−0.134^*^	−0.127^*^	−0.127^*^
Age	−0.009	0.008	0.004	0.020
Education level	−0.002	0.008	0.014	0.016
Working years	0.032	0.007	0.003	−0.012
Organization type	0.073	0.100	0.140	0.123
Occupation type	0.001	0.000	−0.006	−0.012
**Independent variable**				
Citizenship pressure		0.176^***^	0.170^***^	0.197^***^
**Moderating variable**				
Transformational leadership			0.216^***^	0.163^**^
Political skill				−0.199^**^
**Interaction term**				
Citizenship pressure × transformational leadership			0.306^***^	0.247^**^
Citizenship pressure × transformational leadership × political skill				−0.053^*^
*F*	1.097	3.291^**^	6.480^***^	6.957^***^
Δ*F*	1.097	16.115^***^	16.413^***^	6.843^***^
*R* ^2^	0.022	0.074	0.169	0.242
Δ*R*^2^	0.022	0.052	0.095	0.073

* *p < 0.05*,

** *p < 0.01*,

*** *p < 0.001*.

In the regression equation model, gender, age, education level, working years, organization type, and occupation type are first taken as control variables. Then, regression analysis is conducted on the utility of target variables. The specific operational steps are to investigate the effect of citizenship pressure on organizational citizenship performance, the moderating effect of citizenship pressure and transformational leadership in a two-dimensional interaction, and the effect of citizenship pressure, transformational leadership, and political skill in a three-dimensional interaction. Six control variables were introduced into the regression equation in the first phase. The data for the citizenship pressure variable is then entered into the regression equation as the second step. Next, the regression equation included the interaction variables of citizenship pressure and transformational leadership. Fourth, in order to test the research hypothesis, the two-dimensional interaction term of citizenship pressure and transformational leadership, the two-dimensional interaction term of transformational leadership and political skill, and the three-dimensional interaction term of political skill and citizenship pressure and transformational leadership were all entered into the regression equation at the same time. The regression analysis results are shown in Table 5.

**Figure 2:**
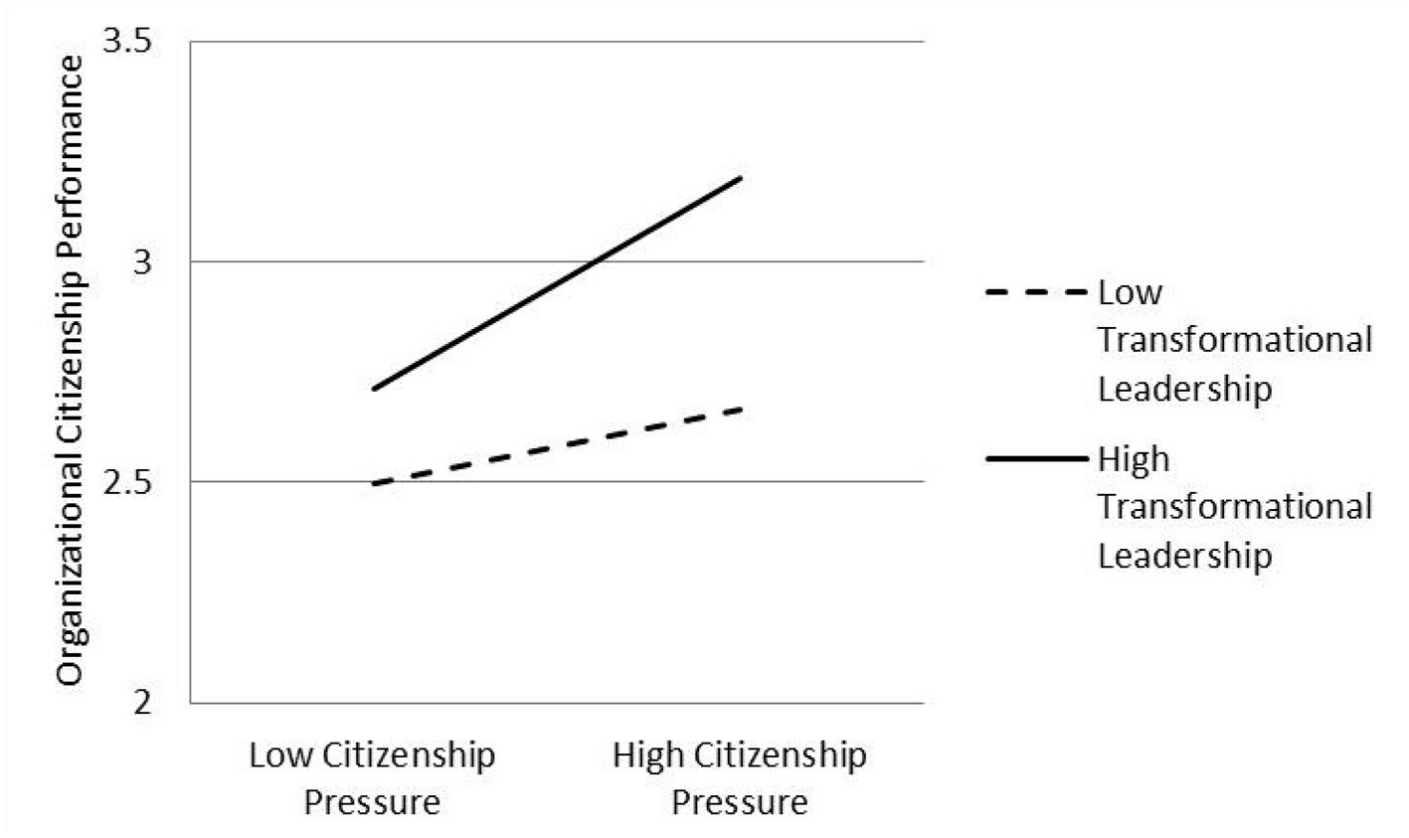
The moderating role of transformational leadership.

After controlling for gender, age, education level, working years, organization type, and occupation type, as well as the main effects of citizenship pressure on organizational citizenship performance, Modle 3 in Table 5 shows that the interaction term of citizenship pressure and transformational leadership has a significant impact on organizational citizenship performance (β = 0.306, *p* < 0.001), which indicates that transformational leadership plays a moderating role, so Hypothesis 2 is supported. Then, Figure 2 is drawn to show the direction and trend of this moderating effect intuitively. Compared with the low transformational leadership situation, citizenship pressure has a more substantial impact on the organizational citizenship performance of employees in the high transformational leadership situation.

Model 4 in Table 5 shows that, after controlling gender, age, education level, working years, organization type, occupation type, citizenship pressure, transformational leadership, political skill, and the interaction term of citizenship pressure × transformational leadership, the three-dimensional interaction term of citizenship pressure, transformational leadership, and political skill significantly impacts the organizational citizenship performance (β = −0.053, *p* < 0.05). Therefore, it can be judged that political skill moderates the effect of transformational leadership on the relationship between citizenship pressure and organizational citizenship performance. The test results initially support Hypothesis 3.

**Figure 3:**
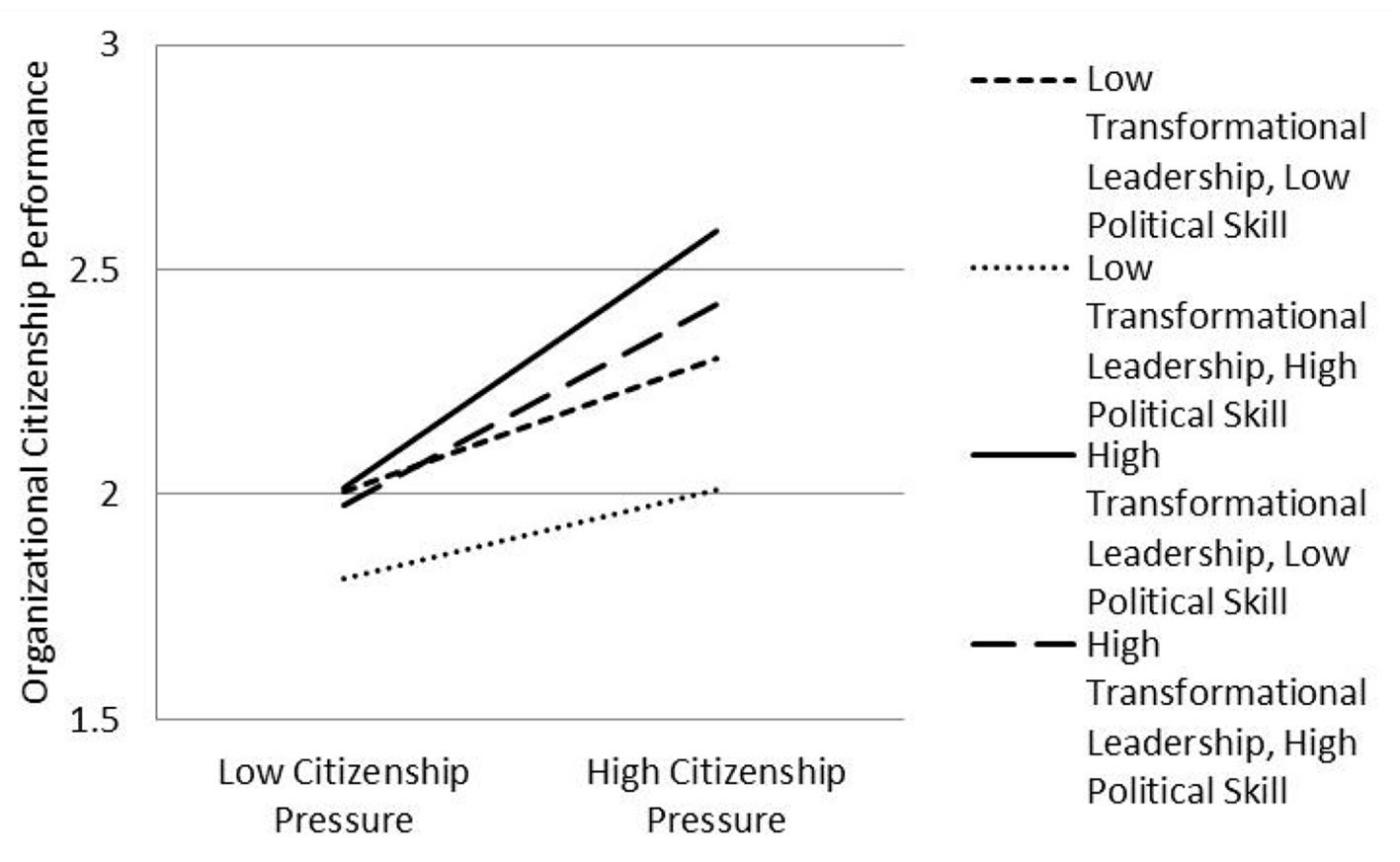
The moderating role of political skill.

In order to better explore the direction and trend of the moderating effect of political skill, the moderating effect diagram of the three-dimensional interaction of “citizenship pressure × transformational leadership × political skill” was drawn in this study. The three-dimensional interaction effect diagram is shown in Figure 3. Organizational citizenship performance is more pertinent in the presence of high transformational leadership and high political skill and high transformational leadership and low political skill than low transformational leadership and high political skill and low transformational leadership and low political skill, indicating that transformational leadership positively moderates the relationship between citizenship pressure and organizational citizenship performance, supporting the Hypothesis 3. In the situation of high transformational leadership, organizational citizenship performance under low political skill is more significant than that under high political skill, indicating that political skill negatively moderates the transformational leadership effect of the relationship between citizenship pressure and citizenship performance, supporting the hypothesis. Thus, transformational leadership plays a positive moderating role in citizenship pressure influencing organizational citizenship performance, whereas, political skill plays a negative moderating role in the moderating effect of transformational leadership. That is, the higher the political skill of employees, the weaker the moderating effect of transformational leadership on the relationship between citizenship pressure and organizational citizenship performance.

## Research Results and Discussion

### Research Results

Based on the expectancy theory, this study explored the mechanism of citizenship pressure influencing organizational citizenship performance and then discussed the moderating role of transformational leadership and political skill. Through the above data analysis, the relationship between the variables is verified and the hypotheses are confirmed: (1) There is a significant positive correlation between citizenship pressure and organizational citizenship performance (*r* = 0.236, *p* < 0.001), which verified Hypothesis 1, that is, citizenship pressure positively affects the organizational citizenship performance of employees; (2) The interaction terms of citizenship pressure and transformational leadership have a significant effect on organizational citizenship performance (β = 0.306, *p* < 0.001), which verified Hypothesis 2, that is, transformational leadership positively moderates the relationship between citizenship pressure and organizational citizenship performance. The stronger the transformational leadership, the more pronounced the positive impact of citizenship pressure on organizational citizenship performance. (3) The three-dimensional interaction of citizenship pressure, transformational leadership, and political skill has a significant effect on organizational citizenship performance (β = −0.053, *p* < 0.05), confirming Hypothesis 3, namely, that the influence of transformational leadership on the relationship between citizenship pressure and organizational citizenship performance is dependent on the political skill of employees. This link between citizenship pressure and organizational citizenship performance gets weak when employees with high political skill face transformational leadership. In contrast, this link is strengthened when employees with low political skill face transformational leadership.

### Theoretical Contributions

First and foremost, this study investigates the process through which citizenship pressure influences organizational citizenship performance, adding to the theoretical viewpoint of citizenship pressure research. Citizenship pressure is a notion derived from the OCB of Western scholars (Bolino et al., 2004). This work has been the first to investigate this link in the Chinese setting to the best of our knowledge. Western experts predicted citizenship pressure based on the history of Western organizations. This research was carried out in Chinese organizations and broadened the area of citizenship pressure research (Cates et al., 2010). We often assume that their OCB would suffer (Bolino and Klotz, 2015). But, contrary to popular belief, we investigate why employees continue to engage in OCBs despite being under pressure. Based on expectancy theory, this study investigates the process through which the citizenship pressure of employees affects their organizational citizenship performance and enhances the theoretical conclusions of current citizenship pressure research by adding empirical data to the outcome variables of citizenship pressure.

Second, the moderating effect of transformational leadership on the link between citizenship pressure and organizational citizenship performance is investigated. This study validates the application of leadership theory in the setting of Chinese organizations. It examines the impact of leadership style on employee behavior in depth. According to existing research, leadership style is the most important element impacting workers, i.e., leadership style may cause employees to feel pressured, which then affects their behaviors (Wu and Peng, 2018). However, according to this study, pressure has a direct impact on the behaviors of employees, and leadership style is the moderating element in this influencing process, which is critical for enriching and developing appropriate organizational theories. In recent years, organizational behavior research has increasingly focused on the work emotions and psychological states of workers, while leadership behavior study has switched from leaders to employees (Podsakoff and Mackenzie, 1997). This research examines leadership style and conduct from the perspective of employees, in order to address this tendency.

Third, earlier conceptualizations of OCB depicted them as discretionary (Organ, 1988). The majority of researches have followed this notion (Xia and Lin, 2021), while the non-discretionary concept is less relevant. There has not been any commensurate empirical development. This study introduces the non-discretionary understanding of OCB, which refers to a behavior that employees are expected and required to engage in to some degree, rather than a behavior that they can choose to engage in (Vigoda-Gadot, 2006). Furthermore, empirical tests were conducted to investigate the impact of citizenship pressure on OCB and the moderating effect of transformational leadership and political skill to broaden our understanding of OCB from various perspectives.

Fourth, because leadership is important for the career growth of workers (Wang and Howell, 2010), this study examines how citizenship pressure (individual perception variable) impacts organizational citizenship performance in the context of transformational leadership. The moderating impact of transformational leadership is influenced by political skill, which symbolizes individual competency elements. By building a positive relationship with transformational leaders (Ferris et al., 2007), employees with high political skill can minimize the expectations and requirements of leaders on them, as well as perceive stresses from leaders as less stressful (Ferris et al., 2007), reducing the occurrence of OCB. The direct impact of political skill on oneself has been the focus of earlier study (Bing et al., 2011). On the contrary, this study explores the influence of political skill on transformational leadership, which can provide self-beneficial outcomes, and broadens the scope of political skill research.

Finally, based on expectancy theory, this study discusses the relationship between citizenship pressure and organizational citizenship performance. Previous studies on stress and performance are more robust and direct research. They divide stress into positive and negative situations to explore the relationship between stress and performance and find that there is a negative impact of stress in both cases, but this study confirms the positive effect of pressure on performance. This study explores the impact of OCB pressure on OCB performance in specific situations and finds that transformational leadership plays a moderating role by encouraging employees, and political skill plays a secondary moderating role by affecting the relationship between leaders and employees. Therefore, this article introduces and verifies the previous theory in the context of OCB pressure, expands the application scope of expectancy theory, promotes the application of expectancy theory in the field of OCB, which is more complex and conditional, and enriches the research on the relationship between pressure and performance.

### Practical Contributions

This study indicates that citizenship pressure may enhance the OCB of employees, as the pressure of employers to promote and raise wages is seen by the employees as a sort of positive pressure. They actively interact with the OCB, which the organization expects, to be driven by the citizenship pressure to meet their goals (Lepine et al., 2005). As a result, we must have a proper knowledge of citizenship pressure to recognize its negative aspects and positive aspects. Proper pressure from citizenship might allow employees to break away and participate in OCBs that benefit the organization. As a result, organizations may use it to improve organizational citizenship performance, establish a citizenship performance appraisal system, formulate corresponding reward and punishment measures, give employees a certain pressure, and encourage employees to regard it as a positive pressure, allowing employees to demonstrate healthy work behaviors while being guided in the correct path.

Second, transformational leaders hope to stimulate the intrinsic motivation of employees through their own personality charm and influence (Bass et al., 1987) to promote employees in an organization and promote the development of the organization. When employees face citizenship pressure, transformational leadership should point them in the right direction, clarify the development prospects and goals of work, stimulate positive expectations in employees for the future, get them to agree on their goals, have expectations for the common vision of enterprises, assist employees in turning pressure into motivation, and avoid employees having negative psychological feelings. Transformational leadership aims to encourage organizational change and attain higher organizational goals, which necessitates communicating and establishing this growth mindset among personnel. Therefore, it is necessary to make clear the promotion path, plan the career path for employees, and draw the blueprint for employees to realize their self-worth to stimulate higher motivation and initiative in employees and then stimulate them to engage in more OCBs.

Third, in some previous studies and practices, the political skill was considered to be unfavorable. Employees with strong political skill, for example, have been found in certain studies to gain promotion by influencing their connection with their supervisors (Liu et al., 2010), which can lead to cliques and is not conducive to organizational fairness. On the contrary, this study shows that political skill of employees may successfully mitigate the detrimental impacts of citizenship pressure. Employees with strong political skill can form good personal connections with their leaders (Ferris et al., 2007), lowering the expectations of their leaders of their OCBs; and they can stay cool and confident while controlling and influencing their environment. They can lessen the negative impact of pressure on them by changing how they perceive and explain the sources of pressure (Ferris et al., 2007). Thus, political skill is beneficial to the individual worker because it can reduce citizenship pressure, which is beneficial to the physical and mental health of employees. However, it is detrimental to the organization because employees with high political skill are less likely to engage in OCB, which is detrimental to the development of an organization. Thus, we cannot simply declare in practice that we need to build the political skill of employees or reduce the political skill of employees, which depends on the demands of organizations.

### Research Limitations and Future Prospects

This study has certain limitations, and it is proposed that it be improved in future studies. In the first place, scales were all translated from foreign scales used in the questionnaire. Although translation decreased the mistake, Chinese companies still have certain restrictions due to disparities between Chinese and international cultural backgrounds. Future studies might look into how the Chinese localization scale could be used to increase the applicability of situations.

Second, data were collected using leader–employee pairs in this study. Employees rated citizenship pressure, transformational leadership, and political skill. In contrast, leaders rated the organizational citizenship performance of employees in order to potentially reduce common method bias. However, because we used single-point data rather than a longitudinal design, We were unable to determine the causation between factors. As a result, future studies can evaluate research hypotheses using multi-point data collection.

Third, this study explores the immediate impact of citizenship pressure on organizational citizenship performance. In the short term, out of reasonable expectations for promotion and salary increase, employees will actively engage in the OCB expected by the organization and leaders. Therefore, citizenship pressure has a significant positive impact on organizational citizenship performance in the short term. But employees involved in too many OCB might lead to time imbalances and temporary and psychological pressure, which let them not to engage in OCB for a longer period of time (Tian and Yang, 2019). Future research might integrate the short- and long-term effects of citizenship pressure on organizational citizenship performance to investigate the curve connection between the two.

Fourth, political skill is a common characteristic that may be expressed through the conduct of organizational citizenship or other conduct (Wu and Yang, 2019). This article nonetheless considers it to be a stable feature of employees and focuses largely on its effect on OCB-O without examining the probable influence of political skill on other organizational behaviors. The links between political skill and other forms of OCB and other organizational behavior might be studied in further research.

Fifth, this study has an organizational context under the impact of Chinese culture. Within the obligatory Chinese culture, the organization and leaders expect employees to get more involved in OCBs (Bolino et al., 2010), forcing employees to do so under this pressure. In other nations with open cultural backgrounds, however, similar phenomena may not occur. In the future, an intercultural study will investigate whether findings may be produced contrary to this work.

## Data Availability Statement

The raw data supporting the conclusions of this article will be made available by the authors, without undue reservation.

## Ethics Statement

The studies involving human participants were reviewed and approved by Hainan University. The patients/participants provided their written informed consent to participate in this study.

## Author Contributions

All authors listed have made a substantial, direct and intellectual contribution to the work, and approved it for publication.

## Funding

This work was supported by the National Social Science Fund of China (21BGL147).

## Conflict of Interest

The authors declare that the research was conducted in the absence of any commercial or financial relationships that could be construed as a potential conflict of interest.

## Publisher's Note

All claims expressed in this article are solely those of the authors and do not necessarily represent those of their affiliated organizations, or those of the publisher, the editors and the reviewers. Any product that may be evaluated in this article, or claim that may be made by its manufacturer, is not guaranteed or endorsed by the publisher.

## References

[B1] Ahearn K. K. Ferris G. R. Hochwarter W. A. Douglas C. Ammeter A. P. (2004). Leader political skill and team performance. J. Manag. 30, 309–327. 10.1016/j.jm.2003.01.004

[B2] Andrews M. C. Kaemar K. M. Harris K. J. (2009). Got Political Skill? The impact of justice on the importance of political skill for job performance. J. Appl. Psychol. 94, 1427–1437. 10.1037/a0017154 19916653

[B3] Avolio B. J. Bass B. M. Jung D. I. (1999). Re-examining the components of transformational and transactional leadership using the multifactor leadership. J. Occup. Organ. Psychol. 72, 441–462. 10.1348/096317999166789

[B4] Avolio B. J. Zhu W. Koh W. Bhatia P. (2004). Transformational leadership and organizational commitment: mediating role of psychological empowerment and moderating role of structural distance. J. Organ. Behav. 25, 951–968. 10.1002/job.283

[B5] Baron R. M. Kenny D. A. (1986). The moderator-mediator variable distinction in social psychological research: conceptual, strategic, and statistical considerations. J. Pers. Soc. Psychol. 51, 1173–1182. 10.1037/0022-3514.51.6.1173 3806354

[B6] Bass B. M. (1985). Leadership performance beyond expectations. Acad. Manage. Rev. 12, 5244–5247.

[B7] Bass B. M. Avolio B. J. (1990). Developing transformational leadership: 1992 and beyond. J. Eur. Ind. Train. 14, 23–42. 10.1108/03090599010135122

[B8] Bass B. M. Waldman D. A. Avolio B. J. Bebb M. (1987). Transformational leadership and the falling dominoes effect. Group Organ. Stud. 12, 73–87. 10.1177/105960118701200106

[B9] Bing M. N. Davison H. K. Minor I. Novicevic M. M. Frink D. D. (2011). The prediction of task and contextual performance by political skill: a meta-analysis and moderator test. J. Vocat. Behav. 79, 563–577. 10.1016/j.jvb.2011.02.006

[B10] Birtch T. A. Chiang F. F. T. Esch E. V. (2016). A social exchange theory framework for understanding the job characteristics—job outcomes relationship: the mediating role of psychological contract fulfillment. Int. J. Hum. Resour. Manag. 27, 1217–1236. 10.1080/09585192.2015.1069752

[B11] Bolino M. C. Hsiung H. H. Harvey J. Lepine J. A. (2015). “Well, I'm Tired of Trying!” Organizational citizenship behavior and citizenship fatigue. J. Appl. Psychol. 100, 56–74. 10.1037/a0037583 25111252

[B12] Bolino M. C. Klotz A. C. (2015). The paradox of the unethical organizational citizen: the link between organizational citizenship behavior and unethical behavior at work. Curr. Opin. Psychol. 6, 45–49. 10.1016/j.copsyc.2015.03.026

[B13] Bolino M. C. Klotz A. C. Turnley W. H. Harvey J. (2013). Exploring the dark side of organizational citizenship behavior. J. Organ. Behav. 34, 542–559. 10.1002/job.1847

[B14] Bolino M. C. Turnley W. H. Gilstrap J. B. Suazo M. M. (2010). Citizenship under Pressure: what's a “Good Soldier” to Do? J. Organ. Behav. 31, 835–855. 10.1002/job.635

[B15] Bolino M. C. Turnley W. H. Niehoff B. P. (2004). The other side of the story: reexamining prevailing assumptions about organizational citizenship behavior. Hum. Resour. Manag. Rev. 14, 229–246. 10.1016/j.hrmr.2004.05.004

[B16] Braendle U. C. Gasser T. Noll J. (2005). Corporate governance in China—Is economic growth potential hindered by Guanxi? Bus. Soc. Rev. 110, 389–405. 10.1111/j.0045-3609.2005.00022.x

[B17] Brislin R. W. (1980). Translation and content analysis of oral and written material, Handbook of Cross-Cultural Psychology: Methodology 2, eds Triandis H. C. Berry J. W. (Boston, MA: Allyn and Bacon), 389–444.

[B18] Burns J. M. (1978). Leadership. New York: Harper and Row.

[B19] Cates D. A. Mathis C. J. Randle N. W. (2010). A positive perspective of citizenship pressure among working adults. J. Manag. Issues 22, 330–344. Available online at: https://www.jstor.org/stable/20798915

[B20] Chen C. H. Liao L. Li Y. Y. Wang T. (2021). Motivation comes from pressure: impact of challenge stressors on individual innovation behavior. Sci. Technol. Prog. Policy. 10.6049/kjjbydc.2020040483. [Epub ahead of print].

[B21] Chen Y. Jia L. Li C. Song J. Zhang J. (2006). Transformational leadership, psychological empowerment, and employee organizational commitment: an empirical study in a chinese context. Manag. World 1, 96–105. 10.19744/j.cnki.11-1235/f.2006.01.012

[B22] Christoph N. Guido H. (2017). Transformational leadership and organizational citizenship behavior: a meta-analytic test of underlying mechanisms. Front. Psychol. 8:364. 10.3389/fpsyg.2017.01364 28848478 PMC5554340

[B23] Douglas C. Ammeter A. P. (2004). An examination of leader political skill and its effect on ratings of leader effectiveness. Leadersh. Q. 15, 537–550. 10.1016/j.leaqua.2004.05.006

[B24] Emerson R. M. (1976). Social exchange theory. Ann. Rev. Soc. 2, 335–362. 10.1146/annurev.so.02.080176.002003

[B25] Farh J. L. Cheng B. S. Chou L. F. Chu X. P. (2006). Authority and benevolence: employees' responses to paternalistic leadership in China, in China's Domestic Private Firms: Multidisciplinary Perspectives on Management and Performance, eds Tsui A. S. Bian Y. Cheng L. (New York, NY: Sharpe), 230–260. Available online at: http://respository.ust.hk/ir/Record/1783.1-12929

[B26] Ferris G. R. Treadway D. C. Kolodinsky R. W. Hochwarter W. A. Kacmar C. J. Douglas C.. (2005). Development and validation of the political skill inventory. J. Manag. 31, 126–152. 10.1177/0149206304271386

[B27] Ferris G. R. Treadway D. C. Perrewe P. L. Brouer R. L. Douglas C. Lux S. (2007). Political skill in organizations. J. Manag. 33, 290–320. 10.1177/0149206307300813

[B28] Graen G. B. Uhl-Bien M. (1995). Relationship-based approach to leadership: development of leader-member exchange theory of leadership over 25 years: applying a muti-level multi-domain perspective. Leadersh. Q. 6, 219–247. 10.1016/1048-9843(95)90036-5

[B29] Howell J. M. Shamir B. (2005). The role of followers in the charismatic leadership process: relationships and their consequences. Aca. Manag. Rev. 30, 96–112. 10.5465/amr.2005.15281435

[B30] Lepine J. A. Podsakoff N. P. Lepine M. A. (2005). A meta-analytic test of the challenge stressor hindrance stressor framework: an explanation for inconsistent relationships among stressors and performance. Aca. Manag. 48, 764–775. 10.5465/amj.2005.18803921

[B31] Liu J. Wu L. Xu J. (2010). The Antecedents and Consequences of the Political Skills: A Longitudinal Case Study. Manag World 11, 94–104. 10.19744/j.cnki.11-1235/f.2010.11.010

[B32] Liu J. G. Zhou Y. Y. Shi Q. W. (2017). Research on Negative Outcomes of Organizational Citizenship Behavior: Based on Generalized Exchange, Impression Management and Evolutionary Psychology. Manag. Rev. 29, 163–180. 10.14120/j.cnki.cn11-5057/f.2017.04.016

[B33] Organ D. W. (1988). Organizational citizenship behavior: the good soldier syndrome. Adm. Sci. Q. 41, 692–703.

[B34] Podsakoff N. P. Whiting S. W. Podsakoff P. M. (2009). Individual-and organizational-level consequences of organizational citizenship behaviors: a meta-analysis. J. Appl. Psychol. 94, 122–128. 10.1037/a0013079 19186900

[B35] Podsakoff P. M. Mackenzie S. B. (1997). The impact of organizational citizenship behavior on organizational performance: a review and suggestions for future research. Human Perform. 10, 133–151. 10.1207/s15327043hup1002_5

[B36] Podsakoff P. M. MacKenzie S. B. Paine J. B. (2000). Organizational citizenship behaviors: a critical review of the theoretical and empirical literature and suggestions for future research. J. Manag. 26, 513–563. 10.1177/014920630002600307

[B37] Schaufeli W. B. Bakker A. B. (2004). Job Demands job resources, and their relationship with burnout and engagement: a multi-sample study. J. Organ. Behav. 25, 293–315. 10.1002/job.248

[B38] Smith C. A. Organ D. W. Near J. P. (1983). organizational citizenship behavior: its nature and antecedents. J. Appl. Psychol. 68, 653–663. 10.1037/0021-9010.68.4.653

[B39] Tian Q. T. Yang Z. W. (2019). The gain and the loss: how does citizenship pressure influence citizenship behavior? Manag. Rev. 31, 163–174. 10.14120/j.cnki.cn11-5057/f.2019.05.015

[B40] Vigoda-Gadot E. (2006). Compulsory citizenship behavior in organizations: theorizing some dark sides of the good soldier syndrome. J. Theory Soc. Behav. 36, 77–93. 10.1111/j.1468-5914.2006.00297.x

[B41] Wang X. H. Howell J. M. (2010). Exploring the dual-level effects of transformational leadership on followers. J. Appl. Psychol. 95, 1134–1144. 10.1037/a0020754 20718529

[B42] Williams L. J. (1991). Job satisfaction and organizational commitment as predictors of organizational citizenship behavior and in-role behavior. J. Manag. 17, 601–617. 10.1177/014920639101700305

[B43] Wu L. W. Yang F. (2019). Political skill: consequences and theoretical explanation. Adv. Psychol. Sci. 27, 163–175. 10.3724/SP.J.1042.2019.02109

[B44] Wu L. Z. Yim F. H. K. Kwan H. K. (2012). Coping with workplace ostraciam: the roles of ingratiation and political skill in employee psychological distress. J. Manag. Stud. 49, 178–199. 10.1111/j.1467-6486.2011.01017.x

[B45] Wu M. Liu Z. Wu J. (2009). The relationship between transformational leadership, psychological empowerment and performance. Soft Sci. 23, 111–117.

[B46] Wu M. Peng Z. (2018). Destructive leadership, supervisor pressure and compulsory organization citizenship behaviors: the moderating role of leader-member exchange. Manag. Rev. 30, 141–152. 10.14120/j.cnki.cn11-5057/f.2018.10.013

[B47] Wu Z. Wu X. (2007). Transformational leadership and organizational citizenship behavior: mediating role of psychological empowerment. J. Manag. Sci. China 10, 40–47. 10.3321/j.issn:1007-9807.2007.05.005

[B48] Xia F. B. Lin Z. (2021). The relationship between organizational citizenship behavior and workplace exclusion——the suppressing effects of envy. Soft Sci. 35, 99–110. 10.13956/j.ss.1001-8409.2021.05.15

[B49] Zhang H. Liu Z. Wang Y. (2020). How transformational leadership positively impacts organizational citizenship behavior in successful Chinese social work service organizations. Non-profit Manag. Leadersh.30, 467–485. 10.1002/nml.21391

[B50] Zhang W. Wang D. (2017). Linking challenge-hindrance stressor to work engagement and counterproductive work behavior. Soft Sci. 31, 75–78. 10.13956/j.ss.1001-8409.2017.11.17

[B51] Zhao H. (2014). Can unwillingness produce desired results?—influence of compulsory citizenship behavior on job performance. Res. Econ. Manag. 11 71–79. 10.13502/j.cnki.issn1000-7636.2014.11.010

[B52] Zhao H. Jiang W. (2017). Citizenship pressure in the workplace. Adv. Psychol. Sci. 25, 312–318. 10.3724/SP.J.1042.2017.00312

[B53] Zhao H. Peng Z. Chen H. (2014). Compulsory citizenship behavior and organizational citizenship behavior: the role of organizational identification and perceived interactional justice. J. Psychol. 148, 177–196. 10.1080/00223980.2013.768591 24684078

[B54] Zhou H. Long L. (2012). The influence of transformational leadership on voice behavior: mediating effect of psychological ownership for the organization and moderating effect of traditionality. Acta Psychol. Sin. 44, 388–399. 10.3724/SP.J.1041.2012.00388

